# Supplementary material for: Phylogenomics of expanding uncultured environmental Tenericutes provides insights into their pathogenicity and evolutionary relationship with Bacilli
Source: BMC Genomics. 2020 Jun 17;21:408. doi: 10.1186/s12864-020-06807-4 (PMC7301438; doi:10.1186/s12864-020-06807-4)
Supplement: Supplementary file 3 — Additional file 3: Figure S1. GC content, completeness and size of Tenericutes. Figure S2. Phylogenetic tree of [FeFe]-hydrogenases and their conserved mitifs. Table S1. Genes and their functions in Fig. 3. [file 12864_2020_6807_MOESM3_ESM.docx]

Figure S1. GC content, completeness and size of Tenericutes

The species for each group of Tenericutes and *Staphylococcales* were listed in supplementary file 1. The completeness of the genomes was evaluated by CheckM. GC content of each 1Kb genome region was calculated and plotted for each Tenericutes group and *Staphylococcales*.


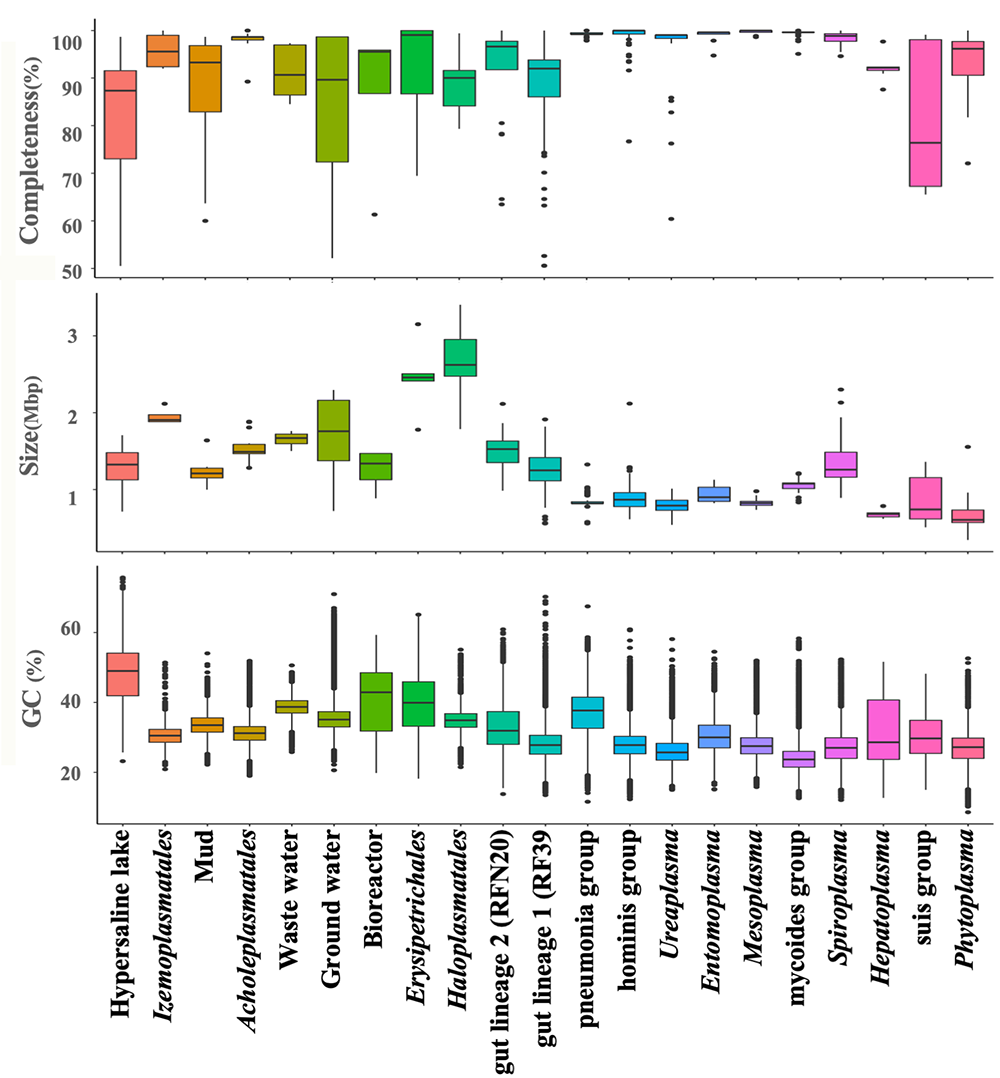


Figure S2. Phylogenetic tree of [FeFe]-hydrogenases and their conserved mitifs.

A maximum-likelihood tree (A) was built with the hydrogenases from RF39 and RNF20 along with the known homologs. The bootstrap values based on 1000 replicates were depicted as dots on the branches (only those >50 were shown). The species names in (B) were labeled on the tree (A) in reference to the accession numbers. The conserved sites of Fe-S cluster and H-cluster in the hydrogenase protein alignment were shaded (B).


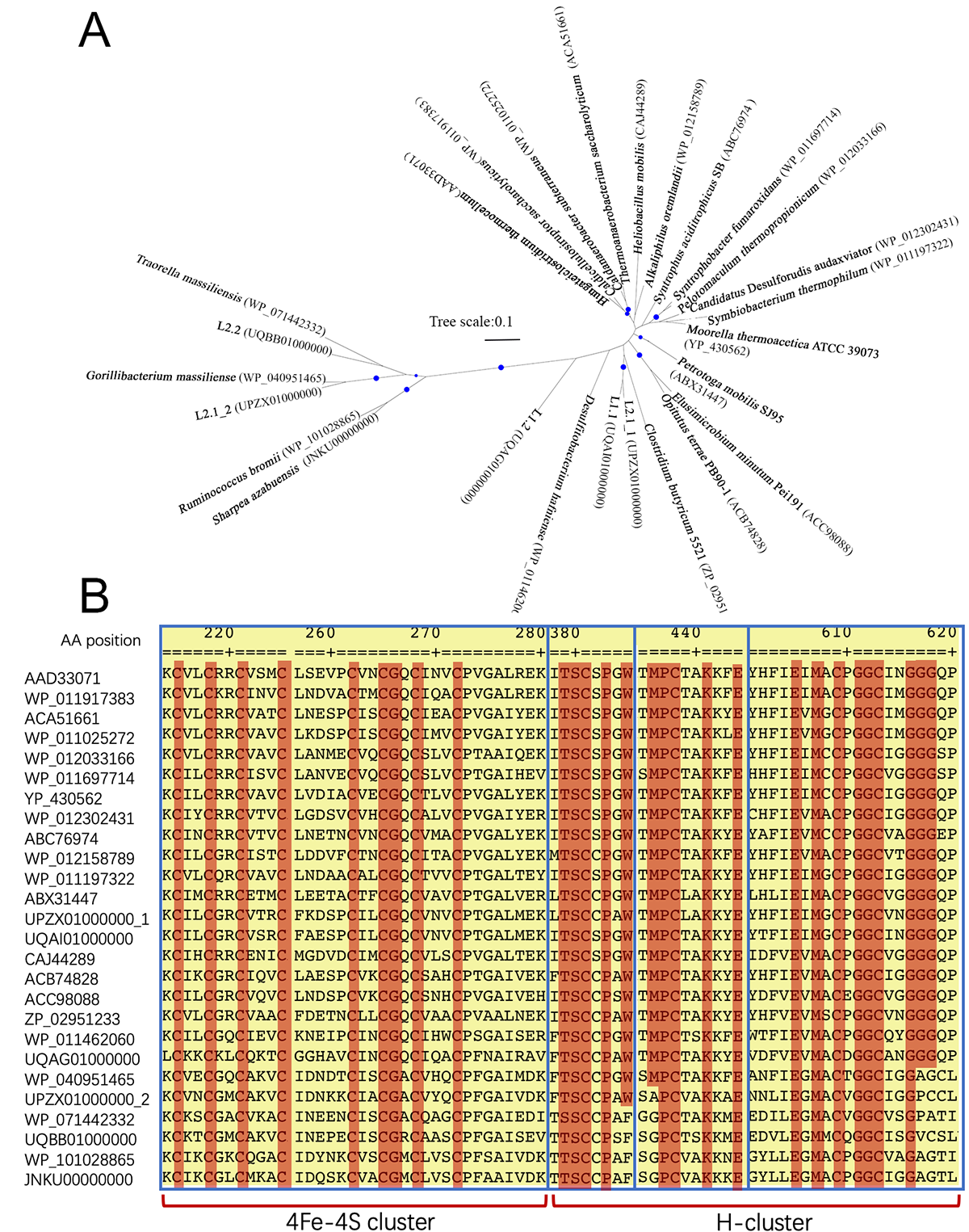


Table S1. Genes and their functions in Figure 3

The gene IDs in COG, KEGG and NOG databases were present.

| ID | gene; function |
| --- | --- |
| COG1070 | xylB; sugar (pentulose and hexulose) kinases |
| COG1940 | nagC; sugar kinase |
| K00845 | glk; glucokinase |
| K00881 | alsK; allose kinase |
| COG0153 | galK; galactokinase |
| K07024 | SPP; sucrose-6-phosphatase |
| K00016 | ldh; lactate dehydrogenase |
| K00161 | pdhA; pyruvate dehydrogenase E1 component alpha subunit |
| COG1180 | pflA; pyruvate-formate lyase-activating enzyme |
| COG1882 | pflD; pyruvate-formate lyase |
| COG4869 | pduL; propanediol utilization protein |
| COG3426 | buk; Butyrate kinase |
| COG0580 | glpF; Glycerol uptake facilitator and related permeases |
| COG0554 | GK; glycerol kinase |
| K00111 | glpA, glpD; glycerol-3-phosphate dehydrogenase |
| K00703 | glgA; starch synthase |
| K00700 | glgB; 1,4-alpha-glucan branching enzyme |
| K00688 | glgP; glycogen phosphorylase |
| K01200 | pulA; pullulanase |
| K00174 | korA; 2-oxoglutarate/2-oxoacid ferredoxin oxidoreductase subunit alpha |
| K01643 | citF; citrate lyase subunit alpha / citrate CoA-transferase |
| K01006 | ppdK; pyruvate, orthophosphate dikinase |
| K00800 | aroA; 3-phosphoshikimate 1-carboxyvinyltransferase |
| K01735 | aroB; 3-dehydroquinate synthase |
| K01736 | aroC; chorismate synthase |
| K01478 | arcA; arginine deiminase |
| K00926 | arcC; carbamate kinase |
| COG0078 | OTC; Ornithine carbamoyltransferase |
| K00605 | gcvT; aminomethyltransferase |
| K02437 | gcvH; glycine cleavage system H protein |
| COG2195 | PepD2; di- and tripeptidases |
| COG3839 | malK; ABC-type sugar transport systems, ATPase components |
| COG3845 | yufO; sugar ABC transporter ATP-binding protein |
| K02765 | PTS-Dgl-EIIC, gamP; D-glucosamine-specific IIC component |
| K02804 | PTS-Nag-EIIC, nagE; N-acetylglucosamine-specific IIC component |
| K02810 | PTS-Scr-EIIC, scrA, sacP,; PTS system, sucrose-specific IIC component |
| K02779 | PTS-Glc-EIIC, ptsG; PTS glucose-specific IIC component |
| K02819 | PTS-Tre-EIIC, treB; PTS trehalose-specific IIC component |
| K02759 | PTS-Cel-EIIA, celC, chbA; PTS cellobiose-specific IIA component |
| K03475 | PTS-Ula-EIIC, ulaA, sgaT; PTS ascorbate-specific IIC component |
| K02770 | PTS-Fru-EIIC, fruA; PTS fructose-specific IIC component |
| K02795 | PTS-Man-EIIC, manY; PTS mannose-specific IIC component |
| K08483 | PTS-EI.PTSI, ptsI; PTS phosphotransferase system, enzyme I |
| K02111 | atpA; F-type H^+^-transporting ATPase subunit alpha |
| K02110 | atpE; F-type H^+^-transporting ATPase subunit c |
| COG4656 | rnfC; NADH:ubiquinone oxidoreductase |
| COG4657 | rnfA; NADH:ubiquinone oxidoreductase RnfA |
| COG4658 | rnfD; NADH:ubiquinone oxidoreductase RnfD |
| K03555 | mutS; DNA mismatch repair protein MutS |
| K03572 | mutL; DNA mismatch repair protein MutL |
| COG2001 | mraZ; cell division/cell wall cluster transcriptional repressor MraZ |
| COG2002 | abrB; regulators of stationary/sporulation gene expression |
| COG1181 | dldA; D-alanine-D-alanine ligase and related ATP-grasp enzymes |
| COG0849 | ftsA; actin-like ATPase involved in cell division |
| COG1077 | mreB; actin-like ATPase involved in cell morphogenesis |
| COG3331 | prfA; required for proper chromosome segregation in *Bacillus subtilis* |
| COG0500 | smtA; SAM-dependent methyltransferases |
| COG0745 | ompR; response regulators with a CheY domain and a winged-helix domain |
| COG0605 | sodA; superoxide dismutase |
| COG0386 | btuE; glutathione peroxidase |
| COG1225 | bcp; peroxiredoxin |
| COG2077 | tpx; peroxiredoxin |
